# Behçet’s disease presenting as neuroretinitis with pre-papillary vitreous infiltrate: a case report

**DOI:** 10.1186/s12348-022-00299-x

**Published:** 2022-07-05

**Authors:** Ahmed Mahjoub, Nadia Ben Abdesslem, Ilhem Sellem, Nesrine Zaafrane, Anis Mahjoub, Fatma Sakji, Mohamed Ghorbel, Hachemi Mahjoub, Wissal Ben Yahia, Ahmad Guiga

**Affiliations:** 1 Department of Ophthalmology, Farhat Hached University Hospital of Sousse, Faculty of Medicine, University of Sousse, Sousse, Tunisia; 2 Department of Internal medicine, Farhat Hached University Hospital of Sousse, Faculty of Medicine, University of Sousse, Sousse, Tunisia

## Abstract

**Objective:**

To report a case of Behçet’s disease (BD) presenting as a panuveitis with neuroretinitis and pre-papillary vitreous infiltrate.

**Material and methods:**

A case report.

**Results:**

A 43-year-old male presented with severely decreased vision in the left eye. Ophthalmological examination revealed unilateral non granulomatous panuveitis with occlusive retinal vasculitis, neuroretinitis and pre-papillary vitreous infiltrate. Initial swept source OCT revealed a “funnel-shaped” hyperreflective lesion overlying the optic disc corresponding to the pre-papillary vitreous infiltrate associated with optic disc edema and a serous retinal detachment extending to the macula. Examination by an internal medicine specialist revealed buccal aphthous ulcer and pseudofolliculitis lesions. BD diagnosis was made and the patient received corticosteroid and immunosuppressive therapy. The pre-papillary vitreous infiltrate resolution under treatment was documented with repeat swept source OCT.

**Conclusion:**

Pre-papillary vitreous infiltrate has been rarely reported in the literature. This finding is a typical feature in severe cases of BD uveitis and is usually associated with a neuroretinitis. Optic disc OCT is useful to make the diagnosis and to monitor the resolution of the pre-papillary vitreous infiltrate.

## Background

Behçet’s disease (BD) is an inflammatory multisystem affection with unknown etiology. It is characterized by the recurrence of oral aphthous ulcers, genital ulcers, skin lesions and uveitis. Ocular involvement seen in BD is often a relapsing non granulomatous uveitis with occlusive vasculitis [1]. Optic nerve involvement in BD may present as papillitis [2], inflammatory optic neuropathy and glaucomatous optic neuropathy [3]. We describe in this report a rare feature of BD uveitis in a patient presenting a neuroretinitis with a pre-papillary vitreous infiltrate, its aspect in swept source optical coherence tomography (SS OCT) and its evolution under treatment.

## Case presentation

**Fig. 1:**
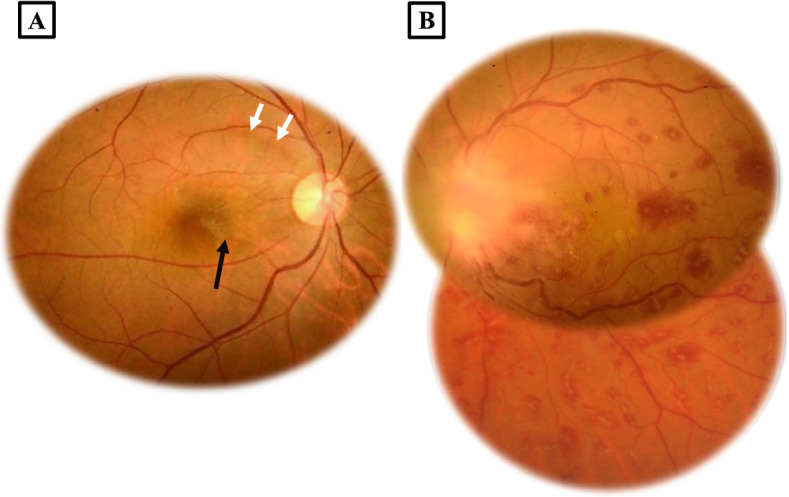
**A** Fundus photograph of the right eye exhibiting an epiretinal membrane (black arrow) and a superotemporal bundle retinal fiber layer defect (white arrows). **B** Fundus photograph of the left eye showing a vitreous opacity overlying the optic nerve head, multiple dot and blot retinal hemorrhages and a vascular sheathing with frosted branch appearance

**Fig. 2:**
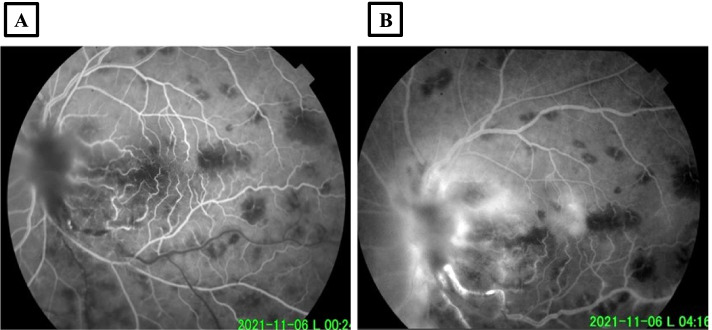
Fundus fluorescein angiography: **A** Early phase revealing delay of filling of the infero-temporal branch retinal vein. **B** Late phase showing hypofluorescence of the optic disc due to the masking effect of vitreous opacity overlying the papillary area, staining of the inferotemporal vein wall as well as vascular leakage of fluorescein dye

**Fig. 3:**
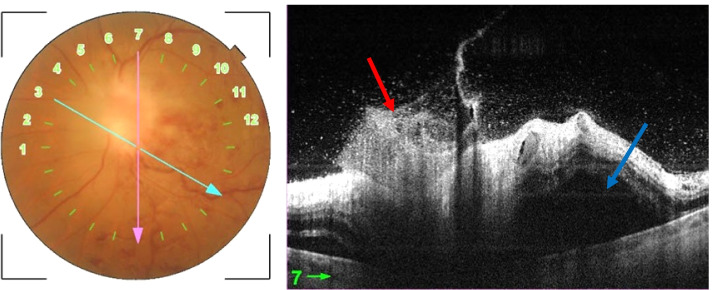
Initial SS OCT scan through the left optic disc exhibiting a hyperreflective lesion in the pre-papillary vitreous area corresponding to the pre-papillary vitreous infiltrate (red arrow) as well as an important optic disc swelling with a large serous retinal detachment extending to the macula (blue arrow)

A 43-year-old immunocompetent Caucasian white male presented with a 3-days history of severely decreased vision in the left eye (LE). He has no past medical history and he described past episodes of red eye self-medicated by steroid eyedrops. On ophthalmological examination, best corrected visual acuity (BCVA) was 20/32 in the right eye (RE) and limited to light perception in the LE. Intra ocular pressures were normal. Slit-lamp examination of the RE did not reveal inflammation in the anterior segment or in the vitreous. Fundus examination showed, an epiretinal membrane and supero-temporal bundle retinal nerve fiber layer defect (RNFL) (Fig. 1A). Slit lamp examination of the LE showed 2+ cells in the anterior chamber and there were no keratic precipitates nor synechiae. Dilated examination of the posterior segment revealed 3+ vitritis and 2+ haze. Fundus examination showed a pre-papillary inflammatory vitreous infiltrate, an important optic disc swelling, multiple dot and blot retinal hemorrhage as well as a vascular sheathing in the infero-temporal retina with frosted branch appearance. (Fig. 1B). Early phase fundus fluorescein angiography (FFA) revealed in the LE an inferotemporal branch retinal vein occlusion (Fig. 2A) and late phase FFA showed a hypofluorescence of the optic disc due to the masking effect of the vitreous opacity overlying the papillary area, staining of the inferotemporal retinal vein and vascular leakage of fluorescein dye (Fig. 2B). SS OCT scan through the optic disc showed a hyperreflective lesion in the pre-papillary vitreous area corresponding to the pre-papillary vitreous infiltrate as well as an important optic disc swelling with a large serous retinal detachment (SRD) extending to the macula (Fig. 3).

At this level, an infectious disease in particular Cytomegalovirus (CMV) retinitis was suspected because of the presence of frosted branch angiitis. The patient was admitted to hospital, intravenous Ganciclovir therapy was introduced and laboratory test were run.

Laboratory testing revealed normal complete blood count, C reactive protein was moderately high (30 mg per litre) and erythrocyte sedimentation rate was high (50 mm the first hour). The patient tested negative for human deficiency virus by ELISA technique, CMV serology, *Treponema pallidum* hemagglutination assay (TPHA) and venereal disease research laboratory (VDRL) and Mantoux test were negative. Chest X ray was normal. General examination by an internal medicine specialist revealed an active oral aphthous ulcer; pseudofolliculitis lesions in the chest area and a history of inflammatory polyarthritis.

The diagnosis of BD was made and a score of 5 as stated by the International Criteria for Behçet’s Disease was attributed. Ganciclovir was discontinued and the patient was treated with 1 g per day of methylprednisolone intravenously (IV) for 3 days followed by oral prednisone at the dosage of 1 mg per kilogram a day associated with azathioprine 150 mg a day and ciclosporin 200 mg a day.

**Fig. 4:**
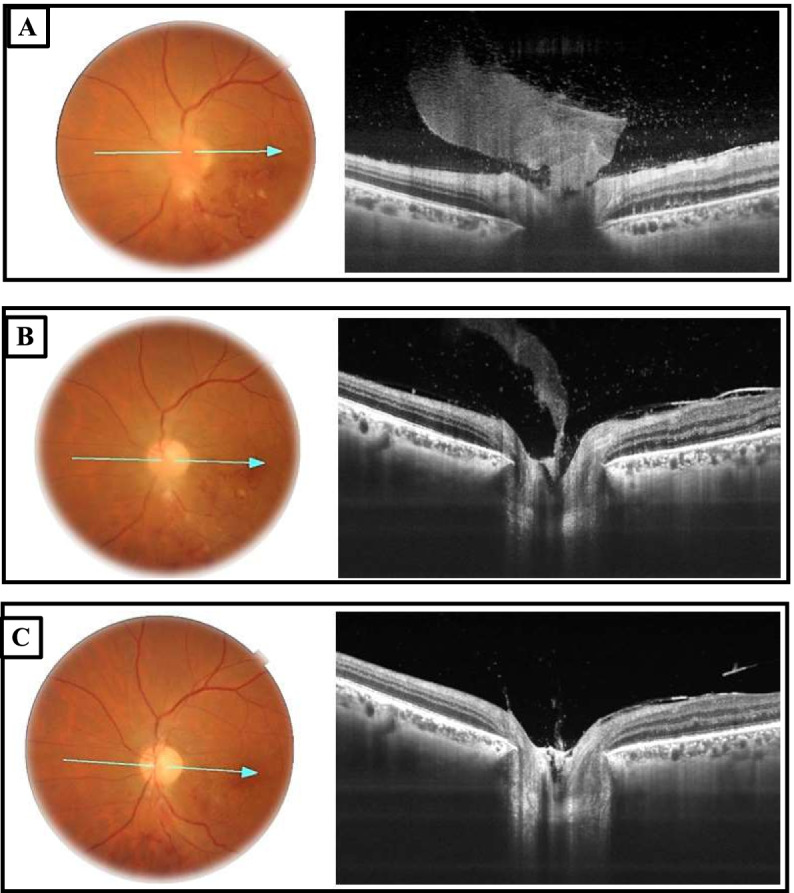
Repeat fundus photographs and optic nerve SS OCT exhibiting the resolution of the pre-papillary vitreous infiltrate under treatment. (**A**) 2 days after the initiation of the treatment, the “funnel shaped” feature of the infiltrate is well-defined on the SS OCT as a hyperreflective lesion in the pre-papillary vitreous and the serous retinal detachment resolved. (**B**) Repeat SS OCT at day 6 showing that the pre-papillary vitreous infiltrate started resolving noticeably. (**C**) Follow-up at day 15, optic disc SS OCT revealing that the pre-papillary vitreous infiltrate resolved completely

After 2 methylprednisolone infusions, the visual acuity in the LE started to improve to counting fingers. Fundus examination exhibited a noticeable decrease of the optic nerve head swelling. SS OCT scan through the optic disc showed a “funnel-shaped” hyperreflective lesion corresponding to the pre-papillary vitreous infiltrate and revealed a resolution of the SRD (Fig. 4A).

Six days after the initiation of treatment, BCVA in the LE improved to 20/400, fundus examination showed a resolution of the vascular sheathing and there were fewer retinal hemorrhages. Optic disc SS OCT revealed that the pre-papillary vitreous infiltrate started resolving noticeably (Fig. 4B).

**Fig. 5:**
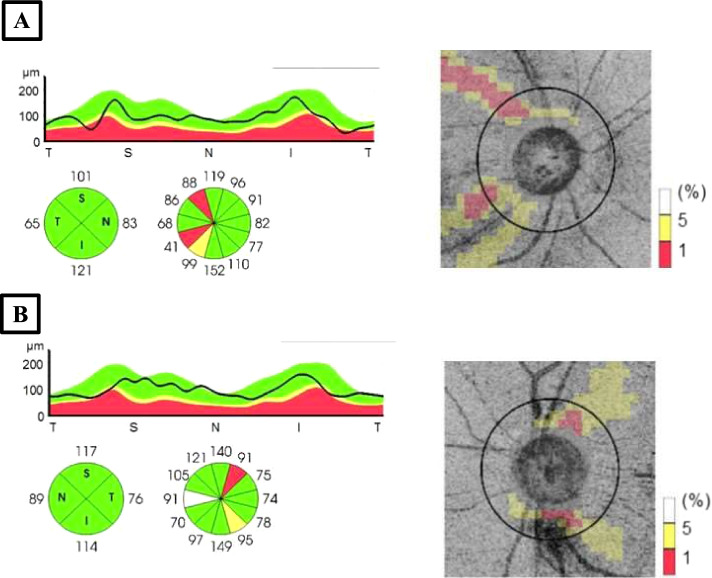
Retinal nerve fibre layer report showing defects in both eyes. **A** right eye; **B** left eye

Follow-up at day 15 revealed temporal pallor of the optic disc, macular hard exudates and fading retinal hemorrhages. SS OCT scan of the optic disc exhibited no pre-papillary vitreous infiltrate (Fig. 4C). Forty-five days later, BCVA remained 20/32 in the RE and improved to 20/63 in the LE. RNFL report showed defects in both eyes suggesting a probable prior unnoticed episode of posterior uveitis in the RE (Fig. 5).

The corticosteroids were tapered progressively and the patient did not have any flare-ups at 6 months follow-up.

## Discussion

Our patient presented a challenging and rare presentation of BD, presenting as a unilateral non granulomatous panuveitis associated to a retinal branch venous occlusion with frosted branch angiitis appearance, neuroretinitis and pre-papillary vitreous infiltrate.

To the best of our knowledge, less than ten cases of BD presenting with pre-papillary vitreous infiltrate have been reported, **(**Table 1**)** with Nakamura et al having described the first case in 2002 [4].

**Table 1:** Summary of the cases of Behçet’s disease uveitis associated with a pre-papillary vitreous infiltrate reported in literature

**Reference**	Age (years)	Sex	Behçet’s disease symptoms	Background treatment	Treatment of the current episode	Time to resolution of the pre-papillary vitreous infiltrate
Nakamura et al. [4]	34	Female	Neuro Behçet’s disease, mucocutaneous lesions and uveitis.	-Prednisolone 25 mg/ day. -Colchicine 1 mg/day.	-Subtenon injection of 0.3 ml dexamethasone. -Augmentation of colchicine dosage to 1.5 mg/day.	11 days.
Schwartz et al. [5]	23	Male	Neuro Behçet’s disease and uveitis.	-Prednisone 12.5 mg/day. -Azathioprine 150 mg/day.	-Augmentation of prednisone dosage to 60 mg/day.	7 days.
Grotting et al. [6]	46	Female	Mucocutaneous lesions and uveitis.	- (Infliximab infusions stopped 1 year before flare-up).	-IV Methylprednisolone 500 mg/day then oral prednisone 60 mg/day.	2 months.
	36	Male	Mucocutaneous lesions and uveitis.	-Prednisone 60 mg/day.	-IV Methylprednisolone 1 g/day. -Sub-tenon injection of Triamcinolone.	4 months.
	26	Male	Pericarditis and uveitis.	-Azathioprine 200 mg/day.	-Prednisone 60 mg/day. -Adalimumab.	3 weeks.
Tugal-Tutkun et al. [7]	37	Female	Uveitis (No additional details).	Not mentioned.	Not mentioned.	6 months.
	35	Female	Uveitis (No additional details).	Not mentioned.	Not mentioned.	3 weeks.
Ksiaa et al. [8]	37	Male	Mucocutaneous lesions and uveitis.	–	-Prednisone 1 mg/kg/day. -Azathioprine 3 mg/kg/day.	1 month.
Our case	43	Male	Mucocutaneous lesions and uveitis.	–	-IV Methylprednisolone 1 g/ per day for 3 days then oral prednisone 1 mg/kg/day. -Azathioprine 150 mg/day. -Ciclosporin 200 mg/day	15 days.

It’s hypothesized that the pre-papillary vitreous infiltrate is the result of the accumulation of polymorphic leucocytes in an enlarged Cloquet canal [4]. The leucocytes may migrate from the optic nerve head infiltration or from an adjacent focus of retinitis or vasculitis [4, 6]. This theory is supported by the “funnel shaped” aspect on OCT of the pre-papillary vitreous infiltrate.

In their review on ocular multimodal imaging in BD, Tugal-Tutkun et al. reported 2 cases of pre papillary vitreous infiltrate and its distinctive characteristic on OCT [7]. In fact, optic disc OCT is a very useful tool in the diagnosis of pre-papillary vitreous infiltrate exhibiting the “funnel-shaped” or “mushroom-shaped” distinctive feature and associated lesions such as serous retinal detachment and optic disc edema. It is also valuable and non-invasive in monitoring the resolution of the vitreous infiltrate under treatment [7, 8].

Early diagnosis and accurate treatment are essential in defining the course of ocular involvement of BD as the structural damage resulting from a severe ocular inflammation can be irreversible and sight-threatening, especially in cases of optic nerve implication [6, 9]. Pre-papillary vitreous infiltrate in cases of BD usually resolve rapidly under corticosteroids. In the published cases, all patients received corticosteroids, either systemically or via subtenon injection. The time to resolution of the vitreous infiltrate is of 7 days [5] to 6 months [7]. In our case, the pre-papillary vitreous infiltrate started resolving by day 6 of corticosteroid treatment and disappeared by day 15 **(**Table 1**)**.

Because of the infrequent reported cases of BD uveitis associated with a pre-papillary vitreous infiltrate, a prognosis correlation could not be established. Nevertheless, previous studies found an association between dense vitreous opacities and poor visual outcomes in BD. It is an indicator of a severe inflammatory reaction which comprises inflammatory mediators such as cytokines and nitric oxide with damaging effects on the neuronal retina [9]. In our case, both eyes exhibited RNFL defects which is a retrospective indicator of previous posterior uveitis attacks in cases of BD [7, 8].

In conclusion, this case indicates that pre-papillary vitreous infiltrate is a rare but typical feature in severe cases of BD uveitis and is usually associated with a neuroretinitis. Multimodal ocular imaging particularly OCT is important to make the diagnosis and to monitor the resolution of the pre-papillary vitreous infiltrate under appropriate treatment.

## Data Availability

All data and supplementary information are available on request.

## Abbreviations

BD: Behçet’s disease
SS OCT: Swept source optical coherence tomography
LE: Left eye
BCVA: Best corrected visual acuity
RE: right eye
RNFL: Retinal nerve fibre layer
FFA: Fundus fluorescein angiography
SRD: Serous retinal detachment
CMV: Cytomegalovirus
TPHA: *Treponema pallidum* hemagglutination assay
VDRL: Venereal disease research laboratory
IV: Intravenous
